# Supplementary material for: Transcutaneous spinal direct current stimulation (tsDCS) does not affect postural sway of young and healthy subjects during quiet upright standing
Source: PLoS One. 2022 Apr 28;17(4):e0267718. doi: 10.1371/journal.pone.0267718 (PMC9049532; doi:10.1371/journal.pone.0267718)
Supplement: S1 File — Translated version of electrical stimulation protocol the assessment form. (DOCX) [file pone.0267718.s001.docx]

**Electrical stimulation assessment**

1. If you felt an electrical stimulation applied by the electrodes positioned on the surface of your body, how long do you think this stimulation lasted (tick only one answer) ?

I felt no electrical stimulation [0]

Very Short [1]

(less than 5 min.)

Short [2]

(5 to 10 min.)

Long [3]

(10 to 15 min.)

Very Long [4]

(more than 15 min.)

1. If you have felt an electrical stimulation, using the scale below, rate the following sensations you may have felt related to the electrical stimulation (tick only one answer for each sensation).

# None = I did not feel it.

**Soft** = I felt it softly.

**Moderate** = I felt it moderately.

**Intense** = I felt it intensely.

**None [0] Soft [1] Moderate [2] Intense [3]**

**Itching**

**Pain**

**Burning**

**Heating**

**Tingling**

**The fields below will be filled in by the researcher:**

Subject ID:____________________________________________________

Protocol ID:

Date:_____/_____/_____

Minor and Major Adverse events:________________________________________

__________________________________________________________________

__________________________________________________________________
