# Supplementary material for: Transcutaneous spinal direct current stimulation (tsDCS) does not affect postural sway of young and healthy subjects during quiet upright standing
Source: PLoS One. 2022 Apr 28;17(4):e0267718. doi: 10.1371/journal.pone.0267718 (PMC9049532; doi:10.1371/journal.pone.0267718)
Supplement: S2 File — Tables of averages and standard deviations of all COP parameters at different stages (t0, t1 and t2) of the three electrical stimulation protocols, quartile ranges of the electrical stimulation assessments answers and results of the statistical analysis both for the COP parameters and for the results of the assessment of electrical stimulation. (PDF) [file pone.0267718.s002.pdf]

Supplementary Information

This file provides the following supplementary information for the paper "Transcutaneous spinal direct current stimulation (tsDCS) does not affect postural sway of young and healthy subjects during quiet upright standing":

- Table of averages and standard deviations of the COP parameters (Table 1).
- Table of the results of the statistical analysis of the COP parameters (Table 2).
- Table of the electrical stimulation protocol group balance for each experimental session (Table 3).
- Table of results obtained from the assessment of the electrical stimulation (Table 4).
- Table of the results of the statistical analysis performed on the outcome of the electrical stimulation assessment (Table 5).

Mean and standard deviation of the COP parameters

Table 1. Mean and standard deviation of the COP parameters obtained at different electrical stimulation stages (t0, t1 and t2) of the three electrical stimulation protocols: Sham, (CT10,Aic) and (AT10,Cic).

	**COP parameter, mean (standard deviation)**								
	**Sham**			**C-T10,AiC**			**A-T10,CiC**		
**Parameter (unit)**	**t0**	**t1**	**t2**	**t0**	**t1**	**t2**	**t0**	**t1**	**t2**
SD_{AP_Ram} (mm)	3.47 (0.78)	3.42 (0.73)	3.92 (1.21)	3.75 (0.89)	3.64 (0.88)	3.78 (1.18)	3.74 (0.96)	3.52 (0.76)	3.46 (0.88)
SD_{AP_Tre} (mm)	1.09 (0.31)	1.03 (0.19)	1.05 (0.25)	1.08 (0.43)	1.10 (0.43)	1.14 (0.53)	0.99 (0.32)	1.07 (0.33)	1.07 (0.31)
SD_{AP} (mm)	3.89 (0.80)	3.80 (0.75)	4.29 (1.22)	4.16 (0.97)	4.05 (0.97)	4.21 (1.31)	4.10 (0.98)	3.92 (0.82)	3.86 (0.90)
SD_{ML_Ram} (mm)	2.00 (0.61)	1.84 (0.56)	2.04 (0.71)	1.87 (0.77)	1.93 (1.14)	2.29 (1.21)	1.80 (0.75)	1.83 (0.76)	1.85 (0.69)
SD_{ML_Tre} (mm)	0.59 (0.18)	0.58 (0.21)	0.59 (0.19)	0.61 (0.24)	0.65 (0.28)	0.68 (0.37)	0.60 (0.26)	0.59 (0.30)	0.62 (0.28)
SD_{ML} (mm)	2.15 (0.63)	2.00 (0.60)	2.20 (0.74)	2.05 (0.81)	2.11 (1.19)	2.47 (1.28)	1.95 (0.79)	1.99 (0.81)	2.02 (0.74)
$MVELO_{AP}$ (mm/s)	5.99 (1.58)	5.61 (1.08)	5.77 (1.29)	5.86 (1.80)	5.80 (1.88)	6.02 (2.22)	5.47 (1.34)	5.54 (1.36)	5.63 (1.45)
$MVELO_{ML}$ (mm/s)	2.86 (0.56)	2.76 (0.57)	2.87 (0.69)	2.90 (0.70)	2.86 (0.79)	3.20 (1.30)	2.76 (0.62)	2.65 (0.65)	2.79 (0.63)
$AREAE$ (mm^2)	230.09 (109.34)	210.72 (103.00)	264.74 (157.49)	247.40 (143.66)	255.01 (224.10)	313.74 (243.35)	225.27 (115.67)	216.69 (105.24)	227.60 (135.66)
$AREA_{Rate}$ (mm$^2/s$)	7.89 (3.11)	7.24 (2.70)	8.63 (3.88)	8.30 (4.15)	8.54 (6.46)	10.38 (7.75)	7.47 (2.96)	7.36 (2.97)	7.59 (3.15)
$PSD\_AREA\_HF_{AP}$ (mm^2)	1.13 (0.74)	0.90 (0.43)	0.99 (0.56)	1.06 (0.90)	1.05 (0.91)	1.15 (1.18)	0.83 (0.58)	0.92 (0.60)	0.95 (0.55)
$PSD\_AREA\_HF_{ML}$ (mm^2)	0.24 (0.12)	0.25 (0.12)	0.25 (0.13)	0.26 (0.15)	0.26 (0.19)	0.34 (0.42)	0.25 (0.14)	0.22 (0.10)	0.27 (0.17)
$PSD\_AREA\_LF_{AP}$ (mm^2)	6.83 (2.85)	6.25 (2.51)	7.18 (4.20)	7.99 (4.72)	7.33 (4.94)	8.82 (7.53)	7.24 (3.52)	6.90 (2.91)	6.98 (4.03)
$PSD\_AREA\_LF_{ML}$ (mm^2)	1.60 (1.72)	1.57 (1.82)	1.88 (2.29)	1.78 (1.98)	2.26 (3.04)	2.66 (2.86)	1.43 (1.69)	1.60 (2.30)	1.77 (2.10)
$f50p\_PSD_{AP}$ (Hz)	0.29 (0.03)	0.28 (0.03)	0.27 (0.04)	0.26 (0.04)	0.28 (0.05)	0.27 (0.04)	0.26 (0.04)	0.27 (0.03)	0.28 (0.04)
$f50p\_PSD_{ML}$ (Hz)	0.36 (0.12)	0.35 (0.11)	0.35 (0.12)	0.36 (0.12)	0.35 (0.12)	0.36 (0.14)	0.38 (0.10)	0.37 (0.10)	0.37 (0.10)
$f80p\_PSD_{AP}$ (Hz)	0.52 (0.09)	0.52 (0.10)	0.50 (0.14)	0.47 (0.14)	0.46 (0.12)	0.46 (0.08)	0.46 (0.13)	0.47 (0.11)	0.48 (0.09)
$f80p\_PSD_{ML}$ (Hz)	0.55 (0.17)	0.54 (0.15)	0.53 (0.17)	0.55 (0.19)	0.53 (0.17)	0.53 (0.18)	0.58 (0.15)	0.55 (0.15)	0.57 (0.16)
ΔTc_{AP} (s)	1.85 (0.95)	1.84 (0.77)	1.98 (1.04)	2.06 (0.88)	1.91 (0.77)	1.94 (0.71)	2.21 (1.49)	1.85 (0.65)	-0.83 (8.60)
ΔTc_{ML} (s)	1.08 (1.63)	1.50 (0.91)	1.39 (1.01)	1.25 (0.96)	1.42 (0.69)	1.54 (0.96)	1.14 (0.66)	1.26 (0.72)	1.29 (0.63)
$\left\langle \Delta X^2_{COP_{AP}} \right\rangle_C$ (mm^2)	20.92 (8.75)	19.28 (7.80)	21.78 (13.43)	24.64 (13.75)	22.57 (15.66)	26.61 (23.96)	22.00 (11.79)	19.95 (8.40)	-7.01 (88.25)
$\left\langle \Delta X^2_{COP_{ML}} \right\rangle_C$ (mm^2)	3.56 (5.75)	4.54 (5.07)	4.77 (5.69)	4.70 (6.04)	5.93 (7.11)	7.51 (8.42)	3.71 (4.26)	4.38 (5.86)	4.93 (5.84)

Table 1 continued from previous page

	COP parameter, mean (standard deviation)								
	Sham			C-T10,AiC			A-T10,CiC		
**Parameter (unit)**	**t0**	**t1**	**t2**	**t0**	**t1**	**t2**	**t0**	**t1**	**t2**
Dl_{AP} (mm^2/s)	0.40 (0.30)	0.34 (0.24)	0.62 (0.49)	0.44 (0.35)	0.37 (0.29)	0.36 (0.36)	0.36 (0.37)	0.41 (0.31)	0.54 (0.45)
Dl_{ML} (mm^2/s)	0.16 (0.15)	0.15 (0.14)	0.19 (0.14)	0.14 (0.16)	0.29 (0.55)	0.30 (0.39)	0.13 (0.19)	0.13 (0.12)	0.16 (0.15)
Ds_{AP} (mm^2/s)	6.85 (3.82)	5.81 (2.13)	6.17 (2.59)	6.95 (4.75)	6.82 (5.22)	7.43 (6.46)	5.73 (3.20)	6.04 (3.16)	6.11 (3.16)
Ds_{ML} (mm^2/s)	1.47 (0.60)	1.46 (0.90)	1.55 (0.87)	1.64 (1.01)	1.77 (1.44)	2.23 (2.43)	1.43 (0.82)	1.38 (0.93)	1.64 (1.08)
α_L_{AP}	0.99 (0.11)	0.99 (0.07)	1.03 (0.10)	1.00 (0.08)	0.99 (0.09)	1.00 (0.09)	1.00 (0.08)	0.97 (0.08)	0.98 (0.10)
α_L_{ML}	1.01 (0.13)	0.97 (0.13)	1.01 (0.11)	0.97 (0.13)	0.93 (0.10)	1.01 (0.10)	0.96 (0.17)	0.99 (0.12)	1.00 (0.12)
α_S_{AP}	1.90 (0.02)	1.90 (0.02)	1.90 (0.02)	1.90 (0.02)	1.90 (0.02)	1.90 (0.04)	1.90 (0.03)	1.91 (0.03)	1.90 (0.03)
α_S_{ML}	1.87 (0.05)	1.88 (0.04)	1.88 (0.04)	1.87 (0.05)	1.88 (0.04)	1.87 (0.06)	1.86 (0.06)	1.87 (0.05)	1.86 (0.05)
CI_{AP}	15.62 (0.82)	15.64 (0.80)	15.53 (1.03)	15.41 (0.83)	15.46 (0.74)	15.61 (1.00)	15.87 (0.77)	15.46 (0.83)	15.62 (0.93)
CI_{APv}	18.30 (0.72)	18.31 (0.58)	18.18 (0.96)	17.97 (0.66)	18.27 (0.57)	18.33 (0.90)	18.40 (0.63)	18.33 (0.43)	18.26 (0.74)
CI_{ML}	14.73 (1.13)	14.64 (1.11)	14.82 (0.91)	14.60 (1.21)	14.13 (1.24)	14.50 (1.40)	14.27 (1.42)	14.38 (1.44)	14.04 (1.73)
CI_{MLv}	18.03 (1.18)	18.00 (1.30)	18.02 (1.34)	17.62 (1.37)	17.32 (1.47)	17.48 (1.44)	17.46 (1.17)	17.60 (1.20)	16.93 (1.67)

Legends: SD = standard deviation parameters. $MVELO$ = mean velocity parameters. $AREA$ = 95% confidence elipse area parameters. PSD_AREA_HF = PSD area between 0.5Hz to 2Hz parameters. PSD_AREA_LF = PSD area between 0.05Hz to 0.5Hz parameters. $f50p\_PSD$ = frequency that encompasses 50% of the PSD area parameters. $f80p\_PSD$ = frequency that encompasses 80% of the PSD area parameters. (ΔTc, $\langle \Delta X^2 \rangle_C$, Dl and Ds) = stabilogram diffusion analysis parameters. (α_L and α_S) = frequency-specific fractal analysis parameters. CI = multi-scale entropy analysis parameters. AP = parameter obtained in the anteroposterior direction. ML = parameter obtained in the mediolateral direction. Ram = parameter obtained from the COP rambling signal. Tre = parameter obtained from the COP trembling signal. v = parameter obtained from the COP velocity signal.

Table 2. Results of the statistical analysis of the COP parameters. Two-way ANOVA with main factors "electrical stimulation protocol" ((CT10, AiC), (AT10, CiC) and Sham) and "electrical stimulation stage" (t0 , t1, t2). Cases where the null hypothesis was rejected ($p \leq 0.05$) are filled in dark gray.

	ANOVA					
	Protocol		Stage		Protocol x Stage	
**Parameter (unit)**	**F**(dF,dF_{error})	**p**	**F**(dF,dF_{error})	**p**	**F**(dF,dF_{error})	**p**
SD_{AP_Ram} (mm)	F(2.00,24.00) 0.42	0.659	F(2.00,24.00) 1.41	0.265	F(4.00,48.00) 1.73	0.16
SD_{AP_Tre} (mm)	F(2.00,24.00) 0.48	0.625	F(2.00,24.00) 0.54	0.589	F(2.02,24.19) 1.64	0.214
SD_{AP} (mm)	F(1.37,16.45) 0.58	0.509	F(2.00,24.00) 1.67	0.21	F(4.00,48.00) 1.44	0.235
SD_{ML_Ram} (mm)	F(2.00,24.00) 0.68	0.517	F(2.00,24.00) 2.25	0.127	F(4.00,48.00) 1.28	0.291
SD_{ML_Tre} (mm)	F(2.00,24.00) 0.79	0.466	F(2.00,24.00) 0.56	0.581	F(1.88,22.61) 0.31	0.722
SD_{ML} (mm)	F(2.00,24.00) 0.72	0.499	F(2.00,24.00) 2.34	0.118	F(4.00,48.00) 1.15	0.345
$MVELO_{AP}$ (mm/s)	F(1.38,16.60) 1.18	0.314	F(2.00,24.00) 1.27	0.298	F(1.84,22.11) 1.56	0.233
$MVELO_{ML}$ (mm/s)	F(2.00,24.00) 1.42	0.26	F(2.00,24.00) 1.87	0.177	F(1.49,17.84) 0.71	0.467
$AREAE$ (mm^2)	F(1.41,16.89) 1.14	0.325	F(2.00,24.00) 2.87	0.076	F(4.00,48.00) 0.74	0.567
$AREA_{Rate}$ (mm$^2/s$)	F(1.40,16.77) 1.53	0.241	F(2.00,24.00) 2.91	0.074	F(4.00,48.00) 0.94	0.45
$PSD\_AREA\_HF_{AP}$ (mm^2)	F(1.27,15.26) 0.99	0.358	F(2.00,24.00) 1.43	0.26	F(2.42,29.06) 2.08	0.135
$PSD\_AREA\_HF_{ML}$ (mm^2)	F(2.00,24.00) 0.83	0.449	F(1.24,14.87) 1.09	0.331	F(1.74,20.94) 0.39	0.653
$PSD\_AREA\_LF_{AP}$ (mm^2)	F(1.18,14.17) 0.88	0.382	F(2.00,24.00) 1.73	0.198	F(4.00,48.00) 0.68	0.608
$PSD\_AREA\_LF_{ML}$ (mm^2)	F(2.00,24.00) 0.65	0.533	F(1.31,15.67) 2.05	0.171	F(1.76,21.07) 0.37	0.667
$f50p\_PSD_{AP}$ (Hz)	F(2.00,24.00) 0.91	0.415	F(2.00,24.00) 0.34	0.714	F(4.00,48.00) 2.79	0.037
$f50p\_PSD_{ML}$ (Hz)	F(2.00,24.00) 0.51	0.606	F(2.00,24.00) 1.23	0.311	F(4.00,48.00) 0.23	0.919
$f80p\_PSD_{AP}$ (Hz)	F(2.00,24.00) 3.29	0.054	F(2.00,24.00) 0.09	0.913	F(4.00,48.00) 0.47	0.757
$f80p\_PSD_{ML}$ (Hz)	F(2.00,24.00) 0.65	0.533	F(2.00,24.00) 2.45	0.107	F(4.00,48.00) 0.45	0.769
ΔTc_{AP} (s)	F(1.04,12.49) 1.12	0.314	F(1.01,12.14) 1.06	0.323	F(1.02,12.24) 1.25	0.287
ΔTc_{ML} (s)	F(1.37,16.40) 0.32	0.645	F(2.00,24.00) 1.24	0.308	F(1.67,20.08) 0.48	0.591
$\left\langle \Delta X^2_{COP_{AP}} \right\rangle_C$ (mm^2)	F(1.32,15.82) 1.44	0.258	F(1.01,12.14) 0.87	0.369	F(1.05,12.63) 1.32	0.275
$\left\langle \Delta X^2_{COP_{ML}} \right\rangle_C$ (mm^2)	F(2.00,24.00) 0.75	0.481	F(2.00,24.00) 3.14	0.062	F(1.92,23.02) 0.44	0.643
Dl_{AP} (mm^2/s)	F(2.00,24.00) 0.55	0.586	F(2.00,24.00) 1.61	0.221	F(4.00,48.00) 1.61	0.186

Table 2 continued from previous page

	ANOVA					
	**Protocol**		**Stage**		**Protocol x Stage**	
**Parameter (unit)**	**F**(dF,dF_{error})	**p**	**F**(dF,dF_{error})	**p**	**F**(dF,dF_{error})	**p**
Dl_{ML} (mm^2/s)	F(2.00,24.00) 1.79	0.189	F(2.00,24.00) 0.86	0.437	F(1.72,20.64) 0.56	0.557
Ds_{AP} (mm^2/s)	F(1.19,14.33) 1.12	0.322	F(2.00,24.00) 0.97	0.393	F(2.26,27.07) 1.59	0.222
Ds_{ML} (mm^2/s)	F(2.00,24.00) 1.40	0.266	F(1.18,14.18) 1.30	0.283	F(1.56,18.67) 0.45	0.597
α_L_{AP}	F(2.00,24.00) 0.88	0.426	F(2.00,24.00) 1.73	0.198	F(4.00,48.00) 0.98	0.427
α_L_{ML}	F(2.00,24.00) 0.74	0.489	F(2.00,24.00) 6.34	0.006	F(4.00,48.00) 2.31	0.071
α_S_{AP}	F(2.00,24.00) 0.48	0.623	F(1.37,16.47) 0.34	0.638	F(1.47,17.67) 0.40	0.615
α_S_{ML}	F(2.00,24.00) 2.73	0.085	F(2.00,24.00) 2.18	0.135	F(4.00,48.00) 0.81	0.528
CI_{AP}	F(2.00,24.00) 0.40	0.674	F(2.00,24.00) 0.34	0.713	F(4.00,48.00) 1.13	0.354
CI_{APv}	F(2.00,24.00) 0.56	0.576	F(2.00,24.00) 0.30	0.741	F(2.77,33.28) 1.66	0.198
CI_{ML}	F(2.00,24.00) 2.28	0.124	F(2.00,24.00) 0.43	0.657	F(4.00,48.00) 1.34	0.27
CI_{MLv}	F(2.00,24.00) 2.38	0.114	F(2.00,24.00) 0.94	0.406	F(2.51,30.12) 1.96	0.15

Legends: Protocol = "electrical stimulation protocol" . Stage = "electrical stimulation stage". Protocol x Stage = Interaction between "electrical stimulation protocol" and "electrical stimulation stage". dF = degrees of freedom. dF_{error} = degrees of freedom error. $F(dF, dF_{error})$ = F-test value. $p = p$ value. SD = standard deviation parameters. $MVELO$ = mean velocity parameters. $AREA$ = 95% confidence elipse area parameters. PSD_AREA_HF = PSD area between 0.5Hz to 2Hz parameters. PSD_AREA_LF = PSD area between 0.05Hz to 0.5Hz parameters. $f50p\_PSD$ = frequency that encompasses 50% of the PSD area parameters. $f80p\_PSD$ = frequency that encompasses 80% of the PSD area parameters. (ΔTc, $\left\langle \Delta X^2 \right\rangle_C$, Dl and Ds) = stabilogram diffusion analysis parameters. (α_L and α_S) = frequency-specific fractal analysis parameters. CI = multi-scale entropy analysis parameters. AP = parameter obtained in the anteroposterior direction. ML = parameter obtained in the mediolateral direction. Ram = parameter obtained from the COP rambling signal. Tre = parameter obtained from the COP trembling signal. v = parameter obtained from the COP velocity signal.

Table 3. Electrical stimulation protocol group balance for each experimental session. Distribution of the number of subjects for each electrical stimulation protocol. As an example, from the first row, 5 participants received the CT10,AiC protocol in the first experimental session, 4 participants received this protocol in the second session, and 4 participants received it in the third session.

**Protocol**	**First session**	**Second session**	**Third session**
CT10,AiC	5	4	4
AT10,CiC	6	4	3
Sham	2	5	6

Table 4. Electrical stimulation assessment results for each evaluated parameter in different electrical stimulation protocols. The Q1 (first quartile) column displays the values that divide the results between the smallest 25% and the largest 75%. Column Q2 (second quartile, or median) shows the values that divide the results between the lowest 50% and the highest 50%. Column Q3 (third quartile, or median) presents the values that divide the results between the lowest 75% and the highest 25%. The parameter "duration of electrical stimulation" was rated on a 5-level scale, with level 0 corresponding to no perception of electrical stimulation, level 1 to a stimulation of very short duration, level 2 a short stimulation, level 3 a long stimulation, and level 4 a stimulation of very long duration. The parameters "itching", "pain", "heating", "burning" and "tingling" were assessed on a 4-level scale, with level 0 corresponding to no perception of the sensation, level 1 a soft sensation, level 2 a moderate sensation and level 3 an intense sensation.

Evaluated Parameter	Electrical Stimulation Protocol	Q1 (level)	Q2 (level)	Q3 (level)
Electrical Stimulation Duration	AT10,CiC	1.75	2.00	3.00
	CT10,AiC	2.00	2.00	2.25
	Sham	1.00	2.00	2.00
Itching	AT10,CiC	0.00	0.50	1.00
	CT10,AiC	0.00	0.00	1.00
	Sham	0.00	0.50	1.00
Pain	AT10,CiC	0.00	1.00	1.00
	CT10,AiC	0.00	0.50	1.00
	Sham	0.00	0.00	1.00
Heating	AT10,CiC	0.00	0.50	1.25
	CT10,AiC	0.00	1.00	2.00
	Sham	0.00	0.00	1.00
Burning	AT10,CiC	1.00	1.50	2.00
	CT10,AiC	1.00	1.00	2.00
	Sham	0.00	1.00	1.00
Tingling	AT10,CiC	0.00	1.00	1.00
	CT10,AiC	0.75	1.50	2.00
	Sham	0.00	0.00	1.00

Table 5. Results of the statistical test of the assessment of the electrical stimulation in the different protocols by the Friedman tests ($p \leq 0.05$) with independent variable "electrical stimulation protocol" and dependent variables: "duration of electrical stimulation", "itching", "pain", "burning", "heating", and "tingling". The table provides the test statistic (χ^2) value ("Chi-square"), degrees of freedom ("df") and the significance level ("Asymp. Sig."). The null hypothesis was not rejected in any situation.

**Evaluated Parameter**	χ^2	**df**	p
Electrical stimulation duration	4.47	2	0.107
Itching	1.18	2	0.554
Pain	1.20	2	0.549
Heating	4.96	2	0.084
Burning	3.65	2	0.161
Tingling	5.63	2	0.060
